# *Cheilolejeunea zhui* (Lejeuneaceae, Marchantiophyta), a new species with moniliate ocelli from Guangxi, China

**DOI:** 10.1002/ece3.9962

**Published:** 2023-03-31

**Authors:** Yu‐Mei Wei, Wen Ye, Boon‐Chuan Ho, Qi‐Ming Tang, AJ Harris

**Affiliations:** ^1^ Guangxi Key Laboratory of Plant Conservation and Restoration Ecology in Karst Terrain Guangxi Institute of Botany, Guangxi Zhuang Autonomous Region and Chinese Academy of Sciences Guilin 541006 China; ^2^ State Key Laboratory of Cellular Stress Biology, School of Life Sciences Xiamen University Xiamen 361102 China; ^3^ Key Laboratory of Ministry of Education for Coastal and Wetland Ecosystems, School of Life Sciences Xiamen University Xiamen 361102 China; ^4^ Singapore Botanic Gardens, National Parks Board 1 Cluny Road Singapore 259569 Singapore; ^5^ South China Botanical Garden, Chinese Academy of Sciences Guangzhou 510650 China

**Keywords:** conservation biology, endangered, liverwort, new section, ocelli, subtropical

## Abstract

A new ocellate liverwort species, *Cheilolejeunea zhui* (Lejeuneaceae), is described from Guangxi, China. The new species is similar to the neotropical *C. urubuensis* in having moniliate ocelli in the leaf lobes and in general appearances but differs in having obliquely spreading leaves, obtuse to subacute leaf apex, thin‐walled leaf cells with distinct trigones, shallowly bifid female bracteole apex, and numerous ocelli in its perianths. Molecular phylogeny of data from three regions (nrITS, *trn*L–F, and *trn*G) confirmed the systematic position of this new species to be sister to *C. urubuensis*, well apart from the remaining members of the genus. Based on morphological and molecular evidence, *Cheilolejeunea* sect. *Moniliocella sect. nov.* is proposed to accommodate *C. urubuensis* and *C. zhui*. The discovery of *C. zhui* represents the fourth known species in *Cheilolejeunea* with linearly arranged ocelli.

## Graphical abstract

A new ocellate liverwort species, *Cheilolejeunea zhui* (Lejeuneaceae), is described from Guangxi, China, based on morphological and molecular evidence. A new section of *Cheilolejeunea* sect. *Moniliocella* sect. nov. is proposed to accommodate *C. urubuensis* and *C. zhui*. The discovery of *C. zhui* represents the fourth known species in *Cheilolejeunea* with linearly arranged ocelli.

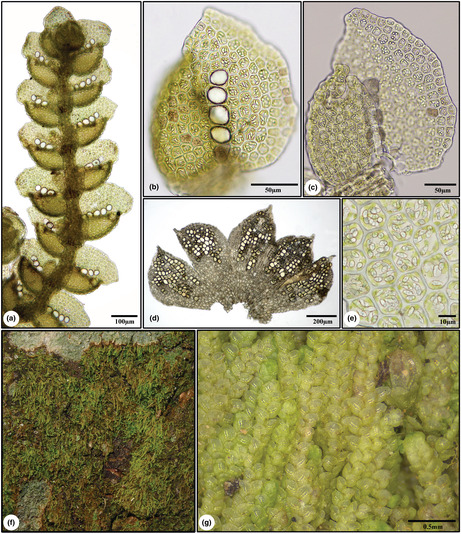

## 1 INTRODUCTION

Guangxi is located in the subtropical region of southern China bordering Vietnam in its southwest. Its heterogeneity in climate, topography, and landscape supports high biodiversity. Bryophyte diversity of Guangxi is among the richest in the country, with over 1230 reported species, representing nearly one‐third of the Chinese bryoflora (Wei et al., 2018; Zhu et al., 2022). Recent bryophyte explorations have led to discoveries of several rare and/or endemic taxa, especially those from Lejeuneaceae, including *Gaolejeunea gaoi* R.L. Zhu & W. Ye (Ye & Zhu, 2018), *Gradsteinianthus tridentatus* (R.L. Zhu et al.) R.L. Zhu & Jian Wang bis, the only species in *Gradsteinianthus* R.L. Zhu & Jian Wang bis (Wang et al., 2016), *Soella obtusifolia* R.L. Zhu, L. Shu, Qiong He & Y.M. Wei (Zhu et al., 2018), and *Vitalianthus guangxianus* R.L. Zhu, Qiong He & Y.M. Wei (He et al., 2012). Interestingly, almost all of these recent records are representatives of oligotypic genera.

**FIGURE 1:**
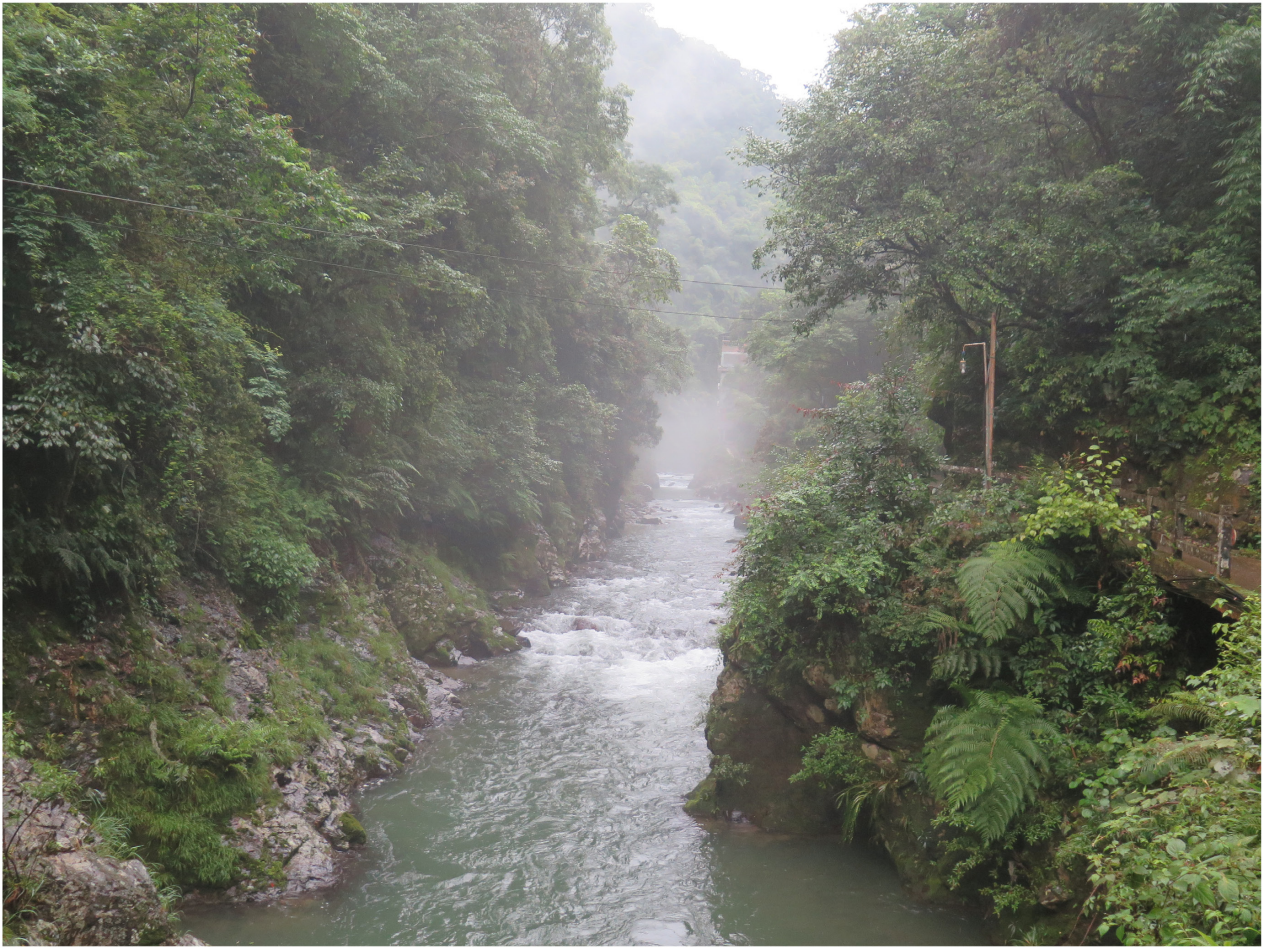
A lowland valley at Guangxi, where *Cheilolejeunea zhui* was found on tree bases and roots along a riverside trail.

During a recent expedition to northern Guangxi, a small population of an unfamiliar species of *Cheilolejeunea* (Spruce) Steph. was encountered (Figure 1). *Cheilolejeunea* is one of the largest and most diverse genera of Lejeuneaceae, with a pantropical distribution (Ye et al., 2015). Recent molecular phylogenetic research on Lejeuneaceae has settled a broader concept of the genus (Schäfer‐Verwimp et al., 2014; Wilson et al., 2007; Ye et al., 2015), which shown that several related genera of Lejeuneaceae, such as *Aureolejeunea* R.M. Schust, *Cyrtolejeunea* A. Evans, *Cystolejeunea* A. Evans, *Evansiolejeunea* Vanden Berghen, *Leucolejeunea* A. Evans, *Omphalanthus* Lindenb. & Nees, and *Trachylejeunea* (Spruce) Steph., are part of and synonymous with *Cheilolejeunea* (Gradstein & Reiner‐Drehwald, 2017; Ye et al., 2015). Therefore, the main key diagnostic features of *Cheilolejeunea* are, the distal hyaline papilla on the leaf lobule, and the leaf cells with few large, coarsely granular (rarely finely granular) oil bodies per cell. The number of accepted species in the genus has reached over 200 (Söderström et al., 2016). However, since a worldwide monograph of *Cheilolejeunea* is still lacking, the actual number of species in the genus may be reduced to an estimated 80–100 species (Bastos & Gradstein, 2020; Ye et al., 2015).

This unusual species that we have encountered along a riverside trail in Guangxi, differs from all described members of *Cheilolejeunea* by having a single row of moniliform ocelli in its leaf lobe, and numerous ocelli in perianth. In this paper, we describe this distinctive species as *Cheilolejeunea zhui*, a new species supported by both morphological and phylogenetic evidence.

## 2 MATERIALS AND METHODS

### 2.1 Morphological observations

The morphological and anatomical characters were examined and photographed using an Olympus SZX7 stereomicroscope and an Olympus BX43 light microscope equipped with a digital camera (Mshot MH125). Field images were taken with a digital camera (Canon G16). Drawing of the voucher specimens was produced using an Olympus BX43 microscope equipped with a drawing tube.

### 2.2 Taxon sampling, DNA extraction, PCR amplification, sequencing, and alignment

We included one freshly collected sample of *Cheilolejeunea zhui* and one sample of *C. urubuensis* (Zartman & I.L. Ackerman) R.L. Zhu & Y.M. Wei (Colombia, *Campos M6‐P*) in our molecular phylogenetic study. Sequences of representatives in the subfamily Ptychanthoideae were selected as an outgroup following the phylogenetic results of Wilson et al. (2007). Sequences of representative species from various subtribes of subfamily Lejeuneoideae were also obtained from GenBank (http://www.ncbi.nlm.nih.gov/genbank/), namely *Ceratolejeunea* (Spruce) J.B. Jack & Steph., *Gaolejeunea* R.L. Zhu & W. Ye, *Lejeunea* Lib., *Leptolejeunea* (Spruce) Steph., and *Pycnolejeunea* (Spruce) Schiffn. Sixty‐one samples from *Cheilolejeunea* representing 50 species and nine out of ten sections of the genus were also included in the dataset (Bastos & Gradstein, 2020; Ye et al., 2015). Voucher information and GenBank accession numbers are presented in Table 1.

**TABLE 1:** List of specimen vouchers and GenBank accession numbers used in this study.

Species	Voucher	ITS	*trnG*	*trn*L–F
*Acrolejeunea fertilis* (Reinw., Blume & Nees) Schiffn.	Indonesia, Schaefer‐Verwimp 17009 (GOET)	DQ987281	JN184493	DQ987391
*Brachiolejeunea laxifolia* (Taylor) Schiffn.	Ecuador, J.A. Shaw 11216E (DUKE)	KT190895	KT190836	KT190779
*Ceratolejeunea coarina* (Gottsche) Schiffn.	Brazil, Zartman 1235.1 (DUKE)	DQ987258	–	DQ238570
*Ceratolejeunea cornuta* (Lindenb.) Steph.	Bolivia, Drehwald 4739 (GOET)	AY607850	AY608170	AY608122
*Cheilolejeunea acutangula* (Nees) Grolle	Brazil, N.D. Santos et al. 400B (RB)	KT190904	KT190845	KT190788
*Cheilolejeunea adnata* (Kunze ex Lehm.) Grolle	Dominica, A. Schäfer‐Verwimp & I. Verwimp 18062 (GOET)	KT190898	KT190839	KT190782
*Cheilolejeunea aurifera* (R.M. Schust.) W. Ye, R.L. Zhu & Gradst.	Costa Rica, I. Holz CR00‐0812 (GOET)	KT190949	KT190883	KT190825
*Cheilolejeunea beyrichii* (Lindenb.) M.E. Reiner I	Honduras, B. Allen 17393 (GOET)	DQ987271	–	DQ987387
*Cheilolejeunea beyrichii* II	Venezuela, T. Pócs 9743/E (EGR)	**–**	**OQ377643**	**OQ377647**
*Cheilolejeunea ceylanica* (Gottsche) R.M. Schust. & Kachroo	China, R.L. Zhu et al. 20050901‐6 (HSNU)	KT190914	KT190852	–
*Cheilolejeunea chenii* R.L. Zhu & M.L. So	China, D.G. Long 33756 (HSNU)	KT190901	KT190842	KT190785
*Cheilolejeunea clypeata* (Schwein.) W. Ye & R.L. Zhu I	U.S.A., B.R. Speer 896 (DUKE)	KT190929	KT190864	KT190808
*Cheilolejeunea clypeata* II	U.S.A., B. Shaw 4714 (DUKE)	KT190928	KT190863	KT190807
*Cheilolejeunea conchifolia* (A. Evans) W. Ye & R.L. Zhu I	U.S.A., P. Majestyk 4927 (DUKE)	KT190925	KT190860	KT190804
*Cheilolejeunea conchifolia* II	U.S.A., B. Shaw 4315 (DUKE)	KT190924	KT190859	KT190803
*Cheilolejeunea cordigera* (Steph.) Grolle	Madagascar, T. Pócs & A. Szabó 9878/FH (EGR)	KT190910	–	KT190794
*Cheilolejeunea ecarinata* Vanden Berghen	Réunion Island, A. Vojtko 9427/AS (EGR)	KT190944	KT190878	–
*Cheilolejeunea falsinervis* (Sande Lac.) R.M. Schust. & Kachroo	China, R.L. Zhu et al. 20031202‐48 (HSNU)	KT190912	–	–
*Cheilolejeunea filiformis* (Sw.) W. Ye, R.L. Zhu & Gradst. I	Puerto Rico, K.M. Pryer 974 (DUKE)	KT190945	KT190879	–
*Cheilolejeunea filiformis* II	Bolivia, S. Churchill et al. 23653 (GOET)	KT190946	KT190880	KT190822
*Cheilolejeunea holostipa* (Spruce) Grolle & R.L. Zhu	Costa Rica, A. Schäfer‐Verwimp & I. Holz SV/H‐0061 (Herb. Schäfer‐Verwimp)	–	KT190841	–
*Cheilolejeunea inflexa* (Hampe ex Lehm.) Grolle	Guadelope, A. Schäfer‐Verwimp & I. Verwimp 22575 (GOET)	KT190906	KT190847	KT190790
*Cheilolejeunea insecta* Grolle & Gradst.	Brazil, A. Schäfer‐Verwimp & I. Verwimp 13447/A (Herb. Schäfer‐Verwimp)	KT190902	KT190843	KT190786
*Cheilolejeunea intertexta* (Lindenb.) Steph.	China, R.L. Zhu et al. 20050908‐20 (HSNU)	KT190908	KT190849	KT190792
*Cheilolejeunea krakakammae* (Lindenb.) R.M. Schust.	China, R.L. Zhu 20070319‐7 (HSNU)	KT190935	KT190869	KT190814
*Cheilolejeunea laevicalyx* (J.B. Jack & Steph.) Grolle I	Mexico, S.R. Gradstein & A. Velasquez s.n. (GOET)	KT190942	KT190876	–
*Cheilolejeunea laevicalyx* II	Ecuador, S.R. Gradstein 10104 (GOET)	KT190941	KT190875	KT190820
*Cheilolejeunea laeviuscula* (Mitt.) Steph.	Thailand, A. Schäfer‐Verwimp & I. Verwimp 23802A (HSNU)	KT190934	KT190868	KT190813
*Cheilolejeunea larsenii* Mizut.	Malaysia, M.S. Chuah & K.T. Yong 09/002/39 (HSNU)	KT190899	KT190840	KT190783
*Cheilolejeunea lineata* (Lehm. & Lindenb.) Schiffn.	Guadeloupe, A. Schäfer‐Verwimp 22183 (GOET)	DQ987295	–	DQ987401
*Cheilolejeunea meyeniana* (Nees, Lindenb. & Gottsche) R.M. Schust. & Kachroo	Australia, E.A. Brown & A. Leishman 2000/45 a (EGR)	KT190915	KT190853	KT190796
*Cheilolejeunea mimosa* (Hook. f. & Taylor) R.M. Schust.	New Zealand, A. Schäfer‐Verwimp & I. Verwimp 13664 (GOET)	KT190943	KT190877	KT190821
*Cheilolejeunea nipponica* (S. Hatt.) S. Hatt.	China, J. Wang et al. 20090801‐5 (HSNU)	KT190909	KT190850	KT190793
*Cheilolejeunea obtusifolia* (Steph.) S. Hatt.	China, R.L. Zhu 20090626‐15 (HSNU)	KT190900	–	KT190784
*Cheilolejeunea occlusa* (Herzog) T. Kodama & N. Kitag.	Indonesia, F.I. Windadri 3876b (IBSC)	KT190911	–	–
*Cheilolejeunea oncophylla* (Ångstr.) Grolle & M.E. Reiner	Dominican Republic, A. Schäfer‐Verwimp & I. Verwimp 26881/A (GOET)	KT190903	KT190844	KT190787
*Cheilolejeunea osumiensis* (S. Hatt.) Mizut.	China, R.L. Zhu 20090220‐25B (HSNU)	KT190931	KT190866	KT190810
*Cheilolejeunea ovalis* (Lindenb. & Gottsche) W. Ye, R.L. Zhu & Gradst. I	Ecuador, A. Schäfer‐Verwimp et al. 24524 (Herb. Schäfer‐Verwimp)	KT190947	KT190881	KT190823
*Cheilolejeunea ovalis* II	Ecuador, A. Schäfer‐Verwimp et al. 24324 (GOET)	KT190948	KT190882	KT190824
*Cheilolejeunea paramicola* (Herzog) W. Ye, R.L. Zhu & Gradst.	Ecuador, A. Schäfer‐Verwimp & M. Preussing 23299/A (Herb. Schäfer‐Verwimp)	KT190952	KT190886	–
*Cheilolejeunea pluriplicata* (Pearson) R.M. Schust.	Thailand, A. Schäfer‐Verwimp & I. Verwimp 23883B (HSNU)	KT190932	KT190867	KT190811
*Cheilolejeunea pocsii* E.W. Jones	Kenya, T. Pócs et al. 04011/AW (EGR)	KT190933	–	KT190812
*Cheilolejeunea quinquecarinata* (R.M. Schust.) W. Ye, R.L. Zhu & Gradst.	Costa Rica, A. Schäfer‐Verwimp & I. Holz SV/H‐0457/A (Herb. Schäfer‐Verwimp)	KT190951	KT190885	–
*Cheilolejeunea rigidula* (Nees ex Mont.) R.M. Schust.	U.S.A., R. Seman 28 (DUKE)	KT190937	KT190871	KT190816
*Cheilolejeunea roccatii* (Gola) W. Ye, R.L. Zhu & Gradst.	Rwanda, E. Fischer X‐RWA‐1120 (Herb. Schäfer‐Verwimp)	KT190802	KT190858	KT190923
*Cheilolejeunea ryukyuensis* Mizut.	China, W. Ye & Y.M. Wei 20090715‐4 (HSNU)	KT190907	KT190848	KT190791
*Cheilolejeunea serpentina* (Mitt.) Mizut.	Madagascar, T. Pócs et al. 90100/AM (EGR)	KT190936	KT190870	KT190815
*Cheilolejeunea sp*	Thailand, A. Schäfer‐Verwimp & I. Verwimp 24032 (HSNU)	KT190905	KT190846	KT190789
*Cheilolejeunea streimannii* Pócs & Ninh	Vietnam, H. Schneider V‐2011‐H‐25‐C (HSNU)	KT190920	–	–
*Cheilolejeunea subopaca* (Mitt.) Mizut.	China, J. Wang & T. Peng 20111018‐48 (HSNU)	KT190921	–	–
*Cheilolejeunea surrepens* (Mitt.) E.W. Jones	Comoro Islands, T. Pócs 9150/W (EGR)	KT190916	KT190854	KT190797
*Cheilolejeunea tonduzana* (Steph.) W. Ye, R.L. Zhu & Gradst.	Brazil, D.P. Costa & S.R. Gradstein 3725 (GOET)	KT190950	KT190884	KT190826
*Cheilolejeunea trapezia* (Nees) Kachroo & R.M. Schust. I	China, W. Ye & Y.M. Wei 20090715‐66 (HSNU)	KT190913	KT190851	KT190795
*Cheilolejeunea trapezia* II	China, R.L. Zhu et al. 20090630‐18 (HSNU)	KT190918	KT190856	KT190799
*Cheilolejeunea trapezia* III	Indonesia, S.R. Gradstein 12057 (GOET)	KT190919	KT190857	KT190800
*Cheilolejeunea trifaria* (Reinw., Blume & Nees) Mizut. I	Seychelles, M.R.D. Seaward 111222 (EGR)	KT190818	KT190873	KT190939
*Cheilolejeunea trifaria* II	Guadelope, A. Schäfer‐Verwimp & I. Verwimp 22434 (GOET)	KT190938	KT190872	KT190817
*Cheilolejeunea trifaria* var. *clausa* (Nees & Mont.) C.J. Bastos & Gradst.	Brazil, N.D. Santos et al. 400A (RB)	KT190940	KT190874	KT190819
*Cheilolejeunea turgida* (Mitt.) W. Ye & R.L. Zhu	China, W. Ye & Y.M. Wei 20090720‐20 (HSNU)	KT190922	–	KT190801
*Cheilolejeunea unciloba* (Lindenb.) Malombe	U.S.A., P. Majestyk 7114 (DUKE)	KT190930	KT190865	KT190809
*Cheilolejeunea urubuensis* (Zartman & I.L. Ackerman) R.L. Zhu & Y.M. Wei	Colombia, L.V.Campos M6‐p (HSNU)	**OQ357714**	**OQ377644**	**OQ377648**
*Cheilolejeunea ventricosa* (Schiffn. ex P. Syd.) X.L. He	China, Ye & Wei 20090715‐53A (HSNU)	**OQ357715**	**OQ377645**	**OQ377649**
*Cheilolejeunea vittata* (Steph. ex G. Hoffm.) R.M. Schust. & Kachroo	China, R.L. Zhu et al. 20050907‐32 (HSNU)	KT190917	KT190855	KT190798
*Cheilolejeunea xanthocarpa* (Lehm. & Lindenb.) Malombe I	China, R.L. Zhu et al. 20090630‐21 (HSNU)	KT190926	KT190861	KT190805
*Cheilolejeunea xanthocarpa* II	Bolivia, S. Churchill 22273 (GOET)	KT190927	KT190862	KT190806
*Cheilolejeunea zhui* Y.M. Wei, W. Ye & B.C. Ho	China, Yu‐Mei Wei 210214‐18A (IBK)	**OQ357716**	**OQ377646**	**OQ377650**
*Gaolejeunea gaoi* (R.L. Zhu, M.L. So & Grolle) R.L. Zhu & W. Ye	China, W. Ye & Y.M. Wei 20090717‐1 (HSNU)	KT190897	KT190838	KT190781
*Lejeunea flava* (Sw.) Nees	U.S.A., J.A. Shaw 9292 (DUKE)	KT190955	KT190888	KT190830
*Leptolejeunea elliptica* (Lehm. & Lindenb.) Besch.	U.S.A., Zona 1531 (HSNU)	MW027044	MW010697	MW010760
*Lopholejeunea nigricans* (Lindenb.) Steph. ex Schiffn.	Malaysia, Gradstein 10357 (GOET)	JN184413	JN184497	JN184541
*Pycnolejeunea densistipula* (Lehm. & Lindenb.) Steph.	Ecuador, Schaefer‐Verwimp 23368 (GOET)	DQ987294	–	DQ987400
*Thysananthus comosus* Lindenb.	Malaysia, Gradstein et al. 10366 (GOET)	DQ987321	JN184523	DQ987425

Accession numbers of newly generated sequences are in bold.

The DNA extraction, PCR, and DNA sequencing methods follow Ye and Zhu (2018). Three molecular genetic makers, nrITS (ITS1‐5.8S‐ITS2), *trn*L–F, and *trn*G were sequenced. Primer references for these regions are given in Ye et al. (2015).

Bidirectional sequences were assembled using PhyDE v.1 (Müller et al., 2010) and then aligned manually in PhyDE v.1 with other sequences of 71 selected Lejeuneaceae species. Ambiguous hotspot positions were excluded prior to molecular phylogenetic analyses; gaps were coded as missing data.

### 2.3 Phylogenetic analyses

Preliminary phylogenetic analyses of the dataset of each marker revealed topological congruence and based on this result they were combined for subsequent analyses. PRAP2 (Müller, 2007) applying the default settings was employed for generating command files using the parsimony ratchet (Nixon, 1999). Maximum Parsimony (MP) analyses were then performed with these command files executed in PAUP* 4.0b10 (Swofford, 2000). Heuristic bootstrap searches under parsimony were performed with 10,000 replicates. JModelTest v.2.1.10 (Darriba et al., 2012) and the corrected Akaike Information Criterion (AICc; Akaike, 1973) were used to select the best‐fit nucleotide substitution for Maximum Likelihood (ML) analysis. This resulted in a Three‐substitution‐type model with unequal base frequencies (TPM1uf), with invariable sites (+I) for the *trn*L–F region; a transversional model (TVM), with gamma‐distributed rate variation (+G) for *trn*G region; and a Transitional model 3 (TIM3), with gamma‐distributed rate variation (+G) and invariable sites (+I) for the nrITS region. MrModeltest v2.4 (Nylander, 2004) was used to establish the best‐fit model for Bayesian inference (BI), and identified a General Time Reversible (GTR) model, with gamma‐distributed rate variation (+G) and invariable sites (+I) as the best‐fit model for the *trn*L–F region and nrITS region, and GTR+ G for the *trn*G region.

The ML trees were generated using the program GARLI version 2.01 (Zwickl, 2006). Best ML trees were found with partitioned analysis based on five independent searches. Bootstrap support (BS) was estimated based on 1070 bootstrap replicates. A partitioning scheme by molecular marker and the same nucleotide substitution models as described above were implemented.

Bayesian inference (BI) was undertaken using MrBayes 3.2.7a (Huelsenbeck & Ronquist, 2001) on the CIPRES Science Gateway (Miller et al., 2010, http://www.phylo.org). The data were partitioned by regions, employing the models suggested by MrModeltest for each partition. Four independent Markov chain Monte Carlo (MCMC) simulations with four chains were run for 10 million generations, sampled every 1000 generations. The program Tracer v1.5 (Rambaut & Drummond, 2009) was used to confirm the burn‐in point and examine the log likelihoods. The outputs from the four runs were combined for the final inference of posterior probabilities (PP) of both trees and model parameters after discarding the burn‐in.

## 3 RESULTS

### 3.1 Morphological observations

Morphological observations in the field showed that fresh plants of *Cheilolejeunea zhui* are glossy light green, leaf lobes with a moniliate, unbroken vitta of ocelli, and underleaves lacking ocelli. Details of the morphological characters of *C. zhui* are treated in the taxonomy sections.

### 3.2 Phylogenetic analyses

**FIGURE 2:**
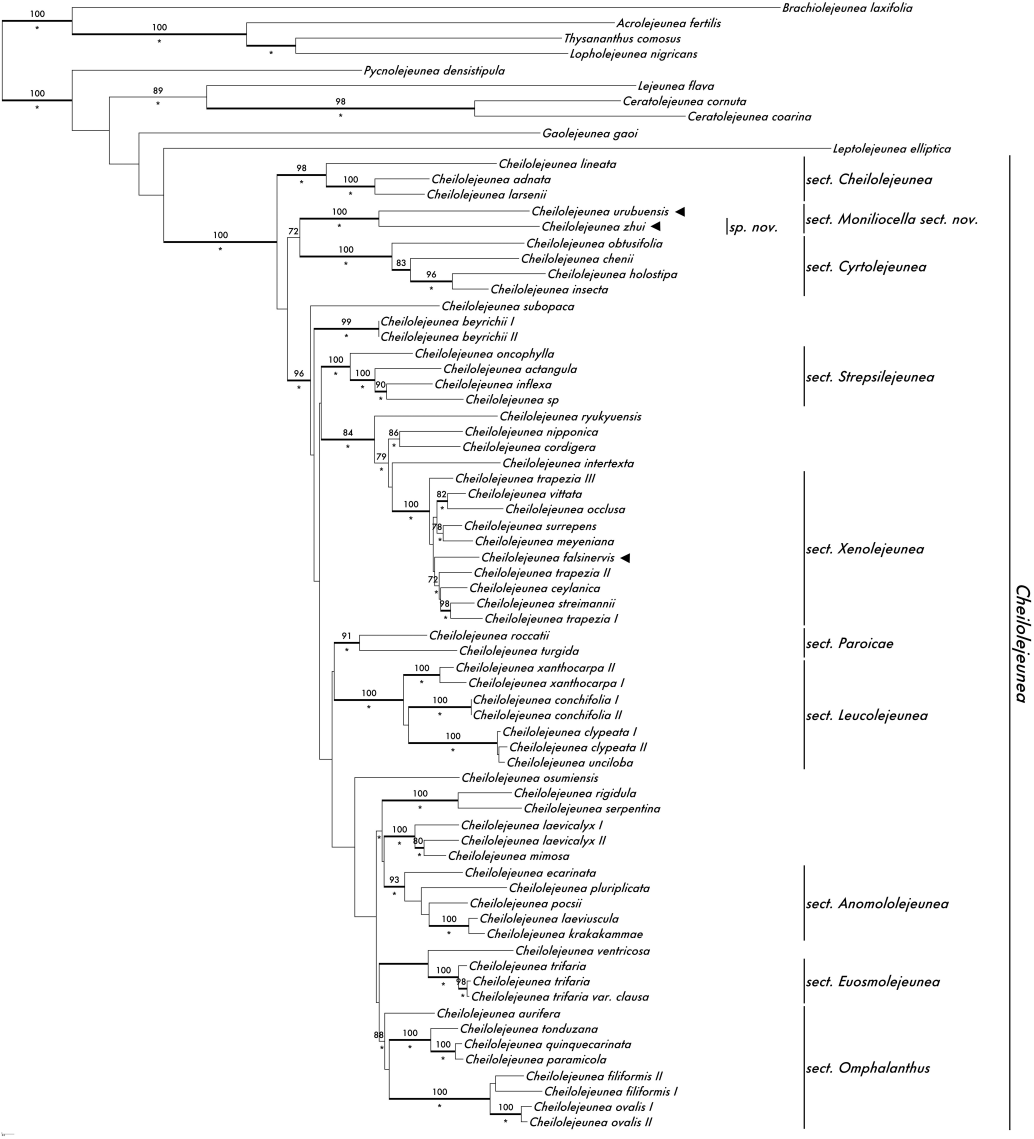
Maximum Likelihood (ML) phylogeny based on *trn*L–F, *trn*G, and nrITS, indicating the position of genus *Cheilolejeunea* sect. *Moniliocella*, and *C. zhui*. Ocellate species included in this study are indicated with “◂”. ML bootstrap values ≥50 are shown above at branches; a star below a branch indicates a Maximum Parsimony bootstrap values ≥75; branches with Bayesian Posterior Probability (BPP) ≥0.95 are in bold line.

The aligned matrix contained 2146 nucleotide characters, including 413 in the *trn*L–F region, 638 in the *trn*G region, and 1095 in the ITS. Of the total character sites, 1076 were constant, 774 were parsimony‐informative and 296 were variable but parsimony uninformative. The MP analysis resulted in 12 maximally parsimonious trees with a length of 4714 steps, with a consistency index (CI) of 0.390 and a retention index (RI) of 0.549, RC of 0.214, and HI of 0.610. All three analyses (BI, ML, and MP) resulted in a largely congruent result. An ML tree with indications of BS calculated in the MP and ML analyses and Bayesian posterior probabilities is given in Figure 2.

*Cheilolejeunea* is reconstructed as monophyletic with maximum support in all the analyses (MLBS 100, MPBS 100, BPP 1). Species with ocelli are found in different lineages of *Cheilolejeunea*. *Cheilolejeunea zhui* is found sister to the neotropical *C. urubuensis* with maximum support (MLBS 100, MPBS 100, BPP 1), together they form a robust lineage sister to sect. *Cyrtolejeunea*. *Cheilolejeunea falsinervis* (Sande Lac.) R.M. Schust. & Kachroo, another species with linear ocelli distributed in China, is grouped with other members of section *Xenolejeunea* (Ye et al., 2015) in a well‐supported clade (MLBS 84, MPBS 89, BPP 1).

## 4 DISCUSSION

The present species clearly belongs to *Cheilolejeunea* in having hyaline papilla at the distal portion of the first tooth of the lobule but differs from other known species of Chinese *Cheilolejeunea* in having numerous ocelli in perianth and moniliate ocelli in the leaf lobe. The new species is most similar to the neotropical *C. urubuensis* in the minute plant size and having an unbroken chain of ocelli (Wei et al., 2013; Zartman & Ackerman, 2002) but is readily separated from the latter in having obliquely spreading leaves, obtuse to subacute leaf apex, thin‐walled leaf cells with distinct trigones, shallowly bifid female bracteole apex, and numerous ocelli in perianths. Moreover, *C. urubuensis* is considered to be a canopy species (Bastos & Gradstein, 2020), while *C. zhui* has been found in the understory so far. Molecular phylogeny also resolves the systematic position of *C. zhui* as sister to *C. urubuensis*, both not belonging to any formal sections of *Cheilolejeunea*.

Ye et al. (2015) classified the species of *Cheilolejeunea* into nine sections based on morphological and molecular evidence: sect. *Anomalolejeunea*, sect. *Cheilolejeunea*, sect. *Cyrtolejeunea*, sect. *Euosmolejeunea*, sect. *Leucolejeunea*, sect. *Paroicae*, sect. *Omphalanthus*, sect. *Strepsilejeunea* and sect. *Xenolejeunea*. A tenth section, sect. *Trachylejeunea* (Spruce) C.J. Bastos & Gradst., was established by Bastos and Gradstein (2020), including seven neotropical species with a pair of closely associated teeth on the lobule and gynoecia frequently without innovations that clearly distinguished them from other sections. Unfortunately, both *Cheilolejeunea urubuensis* and *C. zhui* were unavailable or not yet known for phylogenetic analyses then. Although seemly similar to *C. falsinervis* Kachroo & R.M. Schust. and *C. insignis* Jovet‐Ast & Tixier in having linear form ocelli, *C. urubuensi,s* and *C. zhui* can easily be separated from these members of sect. *Xenolejeunea* by their much smaller plant size, strongly inflated oblong‐ovate lobule, and finely granulated oil bodies. Morphologically and phylogenetically, *C. urubuensis* and *C. zhui* do not fit in any of the formally recognized sections but form a clade sister to sect. *Cyrtolejeunea*. Within *Cheilolejeunea*, *C*. *urubuensis* and *C. zhui* also share the character of minute plant size with sect. *Cheilolejeunea* and sect. *Cyrtolejeunea*, but species from the latter two sections have long to very long, sharp lobule tooth. Therefore, we propose the placement of *C. urubuensis* and *C. zhui* in a new section, sect. *Moniliocella*.

Ocelli, the specialized oil cells filled by a single oil body, are widespread in the family Lejeuneaceae, especially in the subfamily Lejeuneoideae (He & Piippo, 1999). Ocelli are regarded as taxonomically important at generic and specific levels and have evolved more than once in Lejeuneoideae (Dong et al., 2013). Ocelli are usually lacking in *Cheilolejeunea* species, with only a few exceptions (Gradstein, 2013; Zhu et al., 2002), e.g., in *C. aneogyna* (Spruce) A. Evans, *C. insignis*, *C. falsinervis*, *C. urubuensis*, and the present species. The presence of ocelli in *C. aneogyna* is not constant, 2–3 per leaf lobe when present, basal form (Bastos, 2012). Ocelli in other ocellate *Cheilolejeunea* species are arranged in linear form (He & Piippo, 1999), and occur continuously and longitudinally from leaf base to at least the middle of the leaf lobe. In *C. insignis* and *C. falsinervis*, the 1–2(−3) rows of ocelli end near the apex of leaf lobe and extend beyond the length of the adjacent lobule (Jovet‐Ast & Tixier, 1962; Mizutani, 1978). Meanwhile, in *C. urubuensis* and *C. zhui*, ocelli are consistently arranged in a single row, reaching almost the length of the adjacent lobule (see also Zartman & Ackerman, 2002). In our study, ocellate species are found in multiple lineages within *Cheilolejeunea*, indicating that possessing ocelli is a homoplastic character in this genus. The *Cheilolejeunea* species with linear ocelli in leaf lobes are distinguished in the following key.

1. Underleaves with ocelli; known from Borneo, Cambodia, Vietnam .......................... *C. insignis*.

1. Underleaves lacking ocelli; known from tropical Asia, Australasia, and South America ......... 2.

2. Plants larger, more than 0.8 mm wide; stem in transverse section with ca. 10 medullary cells; second tooth on lobule 2–4 cells long; perianths with 5 distinct keels; known from tropical Asia and Australasia ............................................................................ *C. falsinervis*.

2. Plants smaller, less than 0.65 mm wide; stem in transverse section with only 3 medullary cells; second tooth on lobule unicellular; perianths usually with 4 keels ............................................ 3.

3. Leaves spreading from stem at an angle of 60(−80)°; leaf lobe obtuse to subacute at apex; trigones present; female bracteole bilobed to 1/4–1/6 its length at apex; known only from China ........................................................................................................................ *C. zhui*.

3. Leaves spreading from stem at an angle of 80–90°; leaf lobe rounded to rounded‐obtuse at apex; trigones absent; female bracteole entire or weakly emarginate at apex; known from Brazil and Colombia ...................................................... ........ ........ ...... *C. urubuensis*.

## 5 TAXONOMY

### 5.1 ***Cheilolejeunea*** sect. ***Moniliocella*** W. Ye, Y.W. Wei & B.C. Ho, ***sect. nov***.

Type: *C. urubuensis* (Zartman & I.L. Ackerman) R.L. Zhu & Y.M. Wei (≡ *Vitalianthus urubuensis* Zartman & I.L. Ackerman).

Stem without hyalodermis. Leaf apex rounded to subacute. Lobule tooth short or long. Oil bodies several per cell, finely granular. Ocelli present, arranged in a moniliate, unbroken moniliate row form in leaf lobes. Underleaves bifid. Innovations pycnolejeuneoid.

**Etymology**. The epithet refers to the distinctive moniliform longitudinal arrangement of the ocelli in the leaf lobes in both members of the new section.

### 5.2 ***Cheilolejeunea zhui*** Y.M. Wei, W. Ye & B.C. Ho, ***sp. nov.*** (Figures 3 and 4)

**FIGURE 3:**
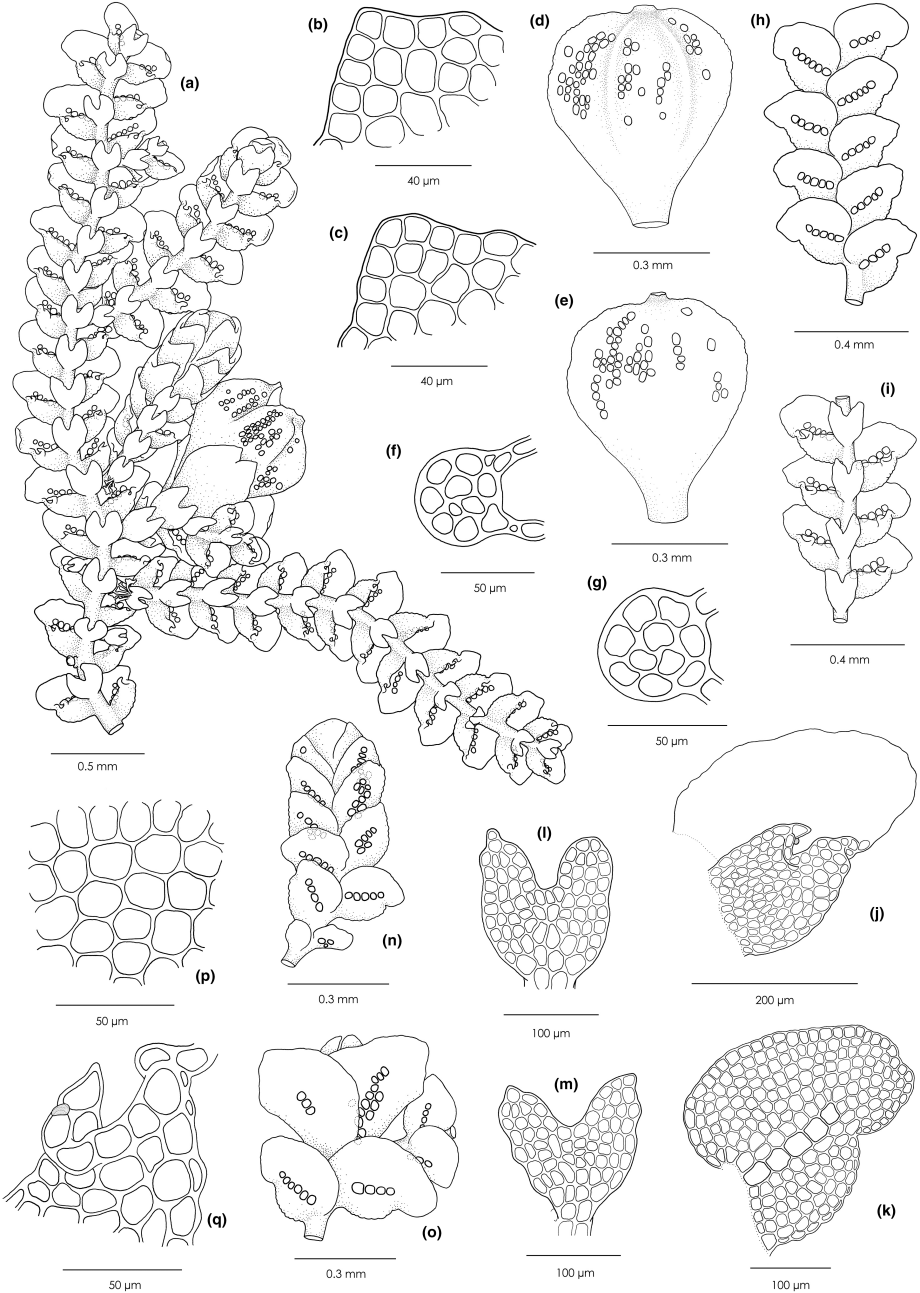
*Cheilolejeunea zhui*. (a) Plants with a perianth and an androecium, ventral view. (b, c) Apices of lateral leaves. (d) Perianth, dorsal view. (e) Perianth, ventral view. (f, g) Cross‐sections of stem. (h) Portion of plant, dorsal view. (i) Portion of plant, ventral view. (j) Leaf, ventral view. (k) Leaf, dorsal view. (l, m) Underleaves. (n) Androecium, dorsal view. (o) Gynoecia with innovation. (p) Median cells of leaf. (q) Apex of leaf lobule. All drawn from *Yu‐Mei Wei 210215–19* (holotype).

**FIGURE 4:**
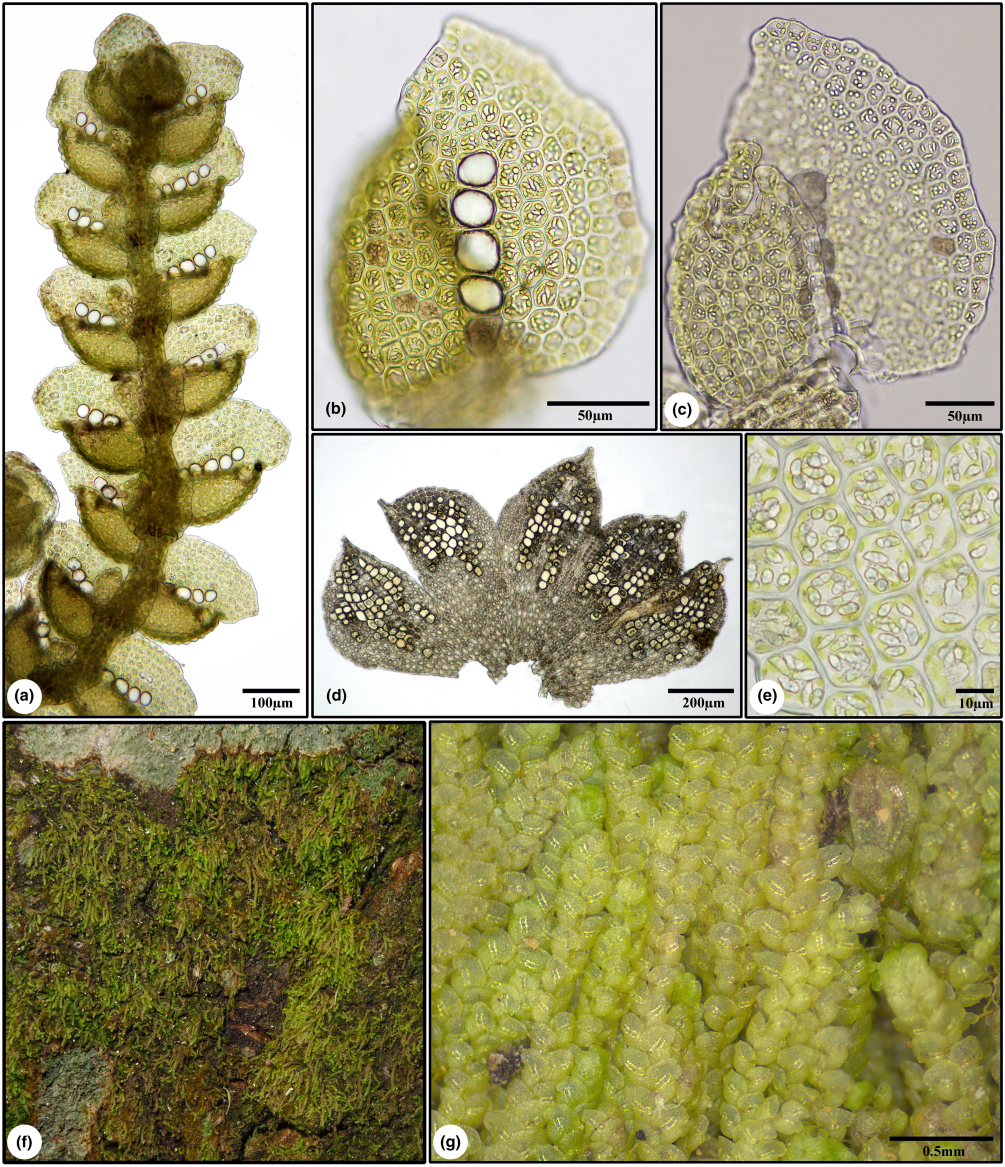
*Cheilolejeunea zhui*. (a) Plant, ventral view. (b) Leaf, dorsal view. (c) Leaf, ventral view. (d) Perianth, opened out. (e) Oil bodies in leaf cells. (f) Population of *C. zhui* on tree base. (g) Living plants of *C. zhui* showing moniliate ocelli. All from *Yu‐Mei Wei 210215–19* (holotype).

Chinese name: 朱氏唇鳞苔.

Diagnosis: *Similar to Cheilolejeunea urubuensis but differs in having obliquely spreading leaves, obtuse to subacute leaf apex, leaf cells with conspicuous trigones, shallowly bifid female bracteole, numerous isolated or aggregated ocelli scattered in perianths*.

Type: CHINA. Guangxi, Longsheng Co., Jiangdi Town, between Longsheng Hot Spring Scenic Spot and Pangxiegou, 110°12″37.59′ E, 25°53″10.42′ N, 301 m, on tree base, mixed with filamentous algae, *Brotherella* sp., and *Syrrhopodon armatus* Mitt., 15 Feb. 2021, *Yu‐Mei Wei 210215–19* (holotype: IBK!, isotypes: AU!, HSNU!, SING!).

Autoicous. Plants light green to yellowish green when fresh, becoming yellow in dried herbarium specimens, up to 15 mm long. Shoots 0.30–0.64 mm wide, irregularly branched, branching of the *Lejeunea*‐type, leaf sequence of vegetative branches lejeuneoid. Stems 43–58 μm in diameter, in transverse section with 7 epidermal cells (13–18 × 11–16 μm) and 3 medullary cells (9–12 × 8–10 μm), both cells slightly thick‐walled; ventral merophyte 2 cells wide. Rhizoids few, hyaline, in tufts from underleaf bases, rhizoid disc absent. Leaves imbricate to contiguous, diverging from stem at an angle of 60(−80)°; leaf lobe ovate, usually convex, slightly falcate, 0.15–0.32 mm long, 0.18–0.26 mm wide, apex obtuse to subacute, usually incurved, margin entire to weakly crenulate, dorsal margin slightly arched; leaf lobule oblong‐ovate, strongly inflated, 3/5–4/5 as long as the lobe, free lateral margin incurved, apex constricted, with 2 teeth, first tooth obsolete, second tooth unicellular, slightly curved toward leaf apex, keel arched, slightly crenulate to crenulate; hyaline papilla spherical, ca. 8 × 6 μm, situated at the distal side of the second tooth. Cells of leaf lobe thin‐walled, trigones small to moderately large, simple‐triangulate, intermediate thickenings occasionally present, at margin quadrate to rectangular, 10–16 × 8–14 μm, in middle subisodiametric to subhexagonal, 13–20 × 9–16 μm, basal cells similar to median ones in shape but slightly larger. Cuticle smooth. Oil bodies of the *Jungermannia*‐type, (4–)6–8(−12) per median cell of leaf lobe. Ocelli (3–)4–5(−7) per leaf, forming an unbroken longitudinal row from leaf base to the middle of the leaf lobe, 20–28 × 18–26 μm, shining and colorless in living material, yellowish‐brown when disintegrated, usually larger than the adjacent cells. Underleaves distant, transversely inserted, 2–3 times as wide as the stem, bilobed to ca. 1/2 underleaf length, the lobes (3–)4–5 cells wide at the base, obtuse to acute at apex, ocelli absent. Androecia terminal on short or long lateral branches, bracts in 3–10 pairs, hypostatic, strongly concave and inflated, shortly and sub‐equally bifid, bract lobe 0.21–0.27 mm long, 0.16–0.19 mm wide, with 3–14 ocelli, ocelli aggregated or forming 1–2 longitudinal, unbroken rows, lobule similar, but slightly shorter, bracteoles 1(−2), present only at the base of the androecium, similar to ordinary underleaves. Gynoecia on main shoots or short lateral branches, with 1 pycnolejeuneoid innovation; bracts 0.33–0.47 mm long, 0.18–0.27 mm wide, bract lobe oblong, apex obtuse to acute, margin entire to weakly crenulate, ocelli vary in numbers, 3–24, suprabasal, isolated, aggregated, or forming 1–2 longitudinal, unbroken rows, bract lobule narrow oblong, 2/3–3/4 as long as the bract lobe, apex acute to acuminate, keel almost straight, ca. 4/5 as long as the lobule; bracteole oblong‐ovate, 0.35–0.40 mm long, 0.22–0.28 mm wide at middle, apex bilobed to 1/4–1/6 its length, margin entire to weakly crenulate. Perianths obovate, 0.63–0.86 mm long, 0.37–0.54 mm wide, with 4 (rarely 5) smooth keels (2 lateral keels, 2 ventral ones in upper 2/3 portion), surface smooth to weakly mammillose, beak 1 cell high, cells rectangular, ocelli numerous, isolated or aggregated, scattered in the upper ventral and dorsal surface. Capsules spherical, longitudinally dehiscing by 4 regular valves at maturity, valves 0.26‐ to 0.30‐mm‐long, 0.16‐ to 0.18‐mm‐wide in the middle. Spores irregularly oblong in shape, 55–84 × 26–35 μm, surface densely papillose, with several rosettes. Elaters attached to the upper 1/3 of each capsule‐valve, nearly hyaline, 170–213 μm long, 11–17 μm wide, strongly sinuately thickened, sometimes with one ± distinct spiral band of thickening. Vegetative reproduction is not seen.

**Etymology**. We dedicate this remarkable species to the Chinese bryologist, Prof. Rui‐Liang Zhu in honor of his outstanding contributions to bryology, especially in the systematics of the family Lejeuneaceae.

**Distribution and ecology**. Known only from the type locality in Longsheng Co., Guangxi, China; epiphytic on tree base and exposed tree root by a trail through the valley in subtropical lowland forest; usually associated with filamentous algae, *Brotherella* sp., *Cololejeunea peraffinis* (Schiffn.) Schiffn., *Leptolejeunea subacuta* Steph. ex A. Evans, and *Syrrhopodon armatus* Mitt.

Longsheng Co., where the new species was discovered, has a subtropical warm and humid climate, with an annual average temperature of 18.4°C, average annual rainfall of 1592 mm, and a frost‐free period of 250–300 days. The warmest temperature of Longsheng is around 37°C, while the lowest temperature is generally −2°C (Writing team of Overview of Longsheng Autonomous County, 2009).

**Provisional IUCN conservation assessment**. The new species has been collected in a habitat under human disturbance along a trail. The small trail serves as the only way to reach nearby villages, as well as leading toward a local scenic spot. The two known small populations, each occupying less than a quarter square meters, would be susceptible to the redevelopment of this trail to cater for heavier human traffic in the future. Although the nearby areas with similar habitats have not been thoroughly surveyed for this species, assessing it as data‐deficient (DD) would be risking the long‐term survival of the existing known populations. This is because there would be little to no conservation afford dedicated to protecting species with a DD status. Following the recommendations of Bergamini et al. (2019) to estimate terricolous taxa occupying an area of one square meter to be equivalent to one mature individual, we are assuming that our vouchers represent a sample of each population. With two isolated “individual‐equivalents,” it fulfills criteria B1 and D of the IUCN Red List (IUCN Standards and Petitions Committee, 2022). As such, we are proposing a provisional conservation status of Critically Endangered (CR) for *Cheilolejeunea zhui* at both national and global levels while waiting for more thorough sampling and surveys in the future to provide more accurate conservation assessments.

**Additional specimens examined**. CHINA. Guangxi, Longsheng Co., Jiangdi Town, between Longsheng Hot Spring Scenic Spot and Pangxiegou, 110°12′25.66″ E, 25°53′19.77″ N, 362 m, on tree root, mixed with *Brotherella* sp., *Cololejeunea peraffinis* (Schiffn.) Schiffn., and *Leptolejeunea subacuta* Steph. ex A. Evans, 14 Feb. 2021, *Yu‐Mei Wei 210214‐18A* (IBK!).

## AUTHOR CONTRIBUTIONS

**Yu‐Mei Wei:** Conceptualization (equal); formal analysis (equal); investigation (lead); resources (lead); visualization (equal); writing – original draft (equal); writing – review and editing (equal). **Wen Ye:** Conceptualization (equal); formal analysis (lead); investigation (equal); resources (equal); visualization (equal); writing – original draft (equal); writing – review and editing (equal). **Boon‐Chuan Ho:** Conceptualization (equal); formal analysis (equal); writing – original draft (equal); writing – review and editing (equal). **Qi‐Ming Tang:** Investigation (equal); resources (equal); visualization (equal); writing – original draft (supporting). **AJ Harris:** Formal analysis (equal); writing – review and editing (equal).

## FUNDING INFORMATION

National Natural Science Foundation of China, Grant/Award Number: 31960045 & 31500170; Guangdong Basic and Applied Basic Research Foundation, Grant/Award Number: 2020A1515010547; Guangxi Natural Science Foundation, Grant/Award Number: 2020GXNSFBA297044.

## CONFLICT OF INTEREST STATEMENT

The authors declare no competing interest.

## Data Availability

The data that newly generated to support the findings of this study are openly available in Genbank at https://www.ncbi.nlm.nih.gov/genbank/, reference number OQ357714‐OQ357716, OQ377643‐OQ377650.
